# Growth and Grazing Kinetics of the Facultative Anaerobic Nanoflagellate, *Suigetsumonas clinomigrationis*

**DOI:** 10.1264/jsme2.ME16113

**Published:** 2017-02-11

**Authors:** Ryuji Kondo, Takahiko Okamura

**Affiliations:** 1 Department of Marine Bioscience, Fukui Prefectural UniversityObama, Fukui, 917–0003Japan; 2 Graduate School of Bioscience and Biotechnology, Fukui Prefectural UniversityObama, Fukui, 917–0003Japan

**Keywords:** anaerobic flagellate, growth rate, ingestion rate, Lake Suigetsu

## Abstract

The functional and numerical responses of the facultative anaerobic heterotrophic nanoflagellate, *Suigetsumonas clinomigrationis* NIES-3647 to prey density were examined under oxic and anoxic conditions. *S. clinomigrationis* grew at temperatures between 10 and 30°C and in the salinity range of 3.9–36.9 psu. The maximum specific growth and ingestion rates of *S. clinomigrationis* were lower under anoxic conditions than under oxic conditions. Half-saturation constants for the growth of *S. clinomigrationis* were within or greater than the range of bacterial densities in the water column of Lake Suigetsu, suggesting that its growth rate is limited by bacterial prey densities in natural environments.

The ‘microbial loop’ is a key component of the planktonic food web in aquatic systems (2, 23). Heterotrophic nanoflagellates (HNF) are important bacterial consumers in the microbial loop (17, 23). HNF have also been detected in the anoxic bottom waters of stratified lakes (11, 18, 21) and in anaerobic cultures from anoxic habitats (3) and deep-sea hydrothermal vents (1). However, HNF ecology in anoxic environments currently remains unclear because of limited data on the abundance and bacterivory of HNF under these conditions (18, 22). We recently detected the high potential bacterivorous activities of HNF in the anoxic layers of meromictic Lake Suigetsu, indicating that anaerobic HNF act as bacteria consumers in the microbial loop of anoxic environments (18).

Understanding of the anaerobic HNF ecology requires knowledge on not only their population dynamics, but also their physiology. However, the physiological characteristics of anaerobic HNF, such as growth and feeding traits, are poorly understood. Physiological properties may provide information on the potential activity of HNF in nature. However, only one study, conducted by Fenchel and Finlay (5), reported the specific growth rate of anaerobic *Hexamita* sp. To the best of our knowledge, detailed studies have yet to be conducted on the bacterivore kinetics of anaerobic HNF. We more recently isolated and described a novel genus and species of facultative anaerobic HNF, *Suigetsumonas clinomigrationis* (stramenopiles, Placididea) from the dissolved-oxygen depleted water just below the oxic–anoxic interface of meromictic Lake Suigetsu (19). The aim of the present study was to assess the numerical (growth rate vs. prey concentration) and functional (ingestion rate vs. prey concentration) responses of this organism under controlled laboratory conditions. The results obtained will contribute to our understanding of the ecological roles of facultative anaerobic HNF in Lake Suigetsu.

We used *S. clinomigrationis* NIES-3647, isolated from the water just below the oxic–anoxic interface of meromictic Lake Suigetsu (19). After the establishment of a monoxenic culture of *S. clinomigrationis* with *Arcobacter* sp., the numerical and functional responses of *S. clinomigrationis* to prey density were examined in batch cultures under oxic and anoxic conditions. Details on the procedures used to assess the eco-physiology of *S. clinomigrationis* are described in Supplementary Materials and Methods.

*S. clinomigrationis* grew within the temperature range of 10–30°C (Fig. 1A) and salinity range of 3.9 to 36.9 psu (Fig. 1B). In Lake Suigetsu, from which the strain was isolated, a meromictic and oxic–anoxic interface developed at a depth of 3–8 m, separating the oxic low-salinity (1–6 psu) mixolimnion from the anoxic saline (12–14 psu) sulphidogenic monimolimnion (10, 14). Water temperatures in the epilimnion change seasonally, ranging from less than 5°C in the winter season to *ca.* 30°C in the summer, while the temperature below the metalimnion is constant at 15°C throughout the year (10, 13, 18). Thus, *S. clinomigrationis* grow in the whole water column of Lake Suigetsu throughout the year, except for the epilimnion in winter. Furthermore, the growth of *S. clinomigrationis* is not restricted by salinity at any depth of the lake. Even in the winter season, the flagellate may be able to grow by escaping from the cool and low-saline water in the epilimnion to the relatively warm water in the metalimnion and hypolimnion.

Two marine species (15, 16) and four halotolerant strains (20) in the class Placididea have been isolated to date. Within the Placididea clade, two environmental clones were detected in Tibetan hypersaline lakes (25). These findings suggest that Placididea flagellates have adapted to marine and/or hypersaline environments. However, even though *S. clinomigrationis* grows in brackish environments, its growth was repressed in full-strength seawater (Fig. 1B). The environmental clone NKS100, which is the most closely related to *S. clinomigrationis* (19), has been retrieved from low saline (9.7 psu) water in an African soda lake (12). Adaptation to low-saline environments appears to be a unique physiological property of *S. clinomigrationis* and the environmental clone NKS100 in the Placididea.

The kinetic values for aerobic HNF vary, and this may be due to inherent differences between species. The values for the maximum specific growth rate (*μmax*) and half-saturation constant (*Kμ*) reported for aerobic HNF are 0.8–6.0 d^−1^ and 0.1–4.5×10^7^ cells mL^−1^, respectively (4 and references therein). The *μmax* of *S. clinomigrationis* as a function of bacterial density, under both aerobic and anaerobic conditions, fit Monod’s equation (Fig. 2). The *μmax_oxi_* (3.4±0.6 d^−1^; mean±SEM) and *Kμ_oxi_* (2.0±1.0×10^7^ cells mL^−1^) of *S. clinomigrationis* under oxic conditions were within these ranges. The *Kμ_anoxi_* (0.6±0.3×10^7^ cells mL^−1^) of the flagellate under anoxic conditions was also within these ranges. However, the *μmax_anoxi_* (0.5±0.0 d^−1^) of *S. clinomigrationis* was approximately 1/7 that under oxic conditions, showing that these conditions are disadvantageous for its growth. The numbers of predators of HNF such as ciliates and metazoan zooplankton are negligible or below the detection limit in the water of the anoxic deeper layer of Lake Suigetsu (9, 18). Therefore, *S. clinomigrationis* may be able to grow and maintain its population in anoxic environments because of the absence of predation pressure. Moreover, if *S. clinomigrationis* is defeated by other HNF in the oxic layer of Lake Suigetsu, the population may be maintained by escaping from the oxic epilimnion to anoxic hypolimnion of the lake.

The ingestion rates of *S. clinomigrationis* as a function of bacterial density fit the Michaelis-Menten hyperbolic relationship between the specific ingestion rate and initial density of *Arcobacter* sp. (Fig. 3). The maximum specific ingestion rate under anoxic conditions (*Imax_anoxi_*: 1.8±0.6 bacteria flagellate^−1^ h^−1^) of *S. clinomigrationis* was approximately 3.5-fold smaller than that under oxic conditions (*Imax_oxi_*: 6.4±0.5 bacteria flagellate^−1^ h^−1^), which had an effect on the lower *μmax_anoxi_*. Despite the low *Imax_anoxi_* by *S. clinomigrationis*, no significant difference was observed between the half-saturation constants (*Ki*) for bacteria ingestion rates under these two conditions (1.6±0.2×10^7^ cells mL^−1^ for oxic conditions and 1.3±1.2×10^7^ cells mL^−1^ for anoxic conditions). *Ki* represents the affinity for phagotrophy and differences in its values reflect changes in the feeding behaviours of phagotrophic flagellates. A transmission electron microscopic examination of the ultrastructures of *S. clinomigrationis* cells indicated that this organism is a raptorial feeder with pseudopod-like structures. No significant difference has been reported between the ultrastructures of cells grown under oxic and anoxic conditions (19); this is reflected by the kinetic values for ingestion.

The growth rates of protists and metazoan zooplankton depend on their individual cell sizes, as shown in Fig. S1 (8). We plotted maximum growth rates as a function of cell volume calculated from the mean cell length and width. The maximum specific growth rate of *S. clinomigrationis* under oxic conditions was within the range of similarly sized aerobic nanoflagellates (Fig. S1). In contrast, under anoxic conditions, the *S. clinomigrationis* growth rate was lower than those of other aerobic nanoflagellates (Fig. S1). The low growth rate observed under anoxic conditions may be explained by the low efficiency of energy production during anaerobic respiration. We calculated gross growth efficiency (*Y*) as follows: *Y*=(*μ*×*V_predator_*)/(*I*×*V_prey_*) (6), where *μ* is the specific growth rate, *V_predator_* and *V_prey_* are the cell volumes of the predator (HNF) and prey (bacteria), respectively, and *I* is the specific ingestion rate of the predator. The carbon content per unit volume of the flagellate and bacterial cells were not taken into account in this calculation. By assuming that *V_predator_* (*S. clinomigrationis*) and *V_prey_* (*Arcobacter* sp.) did not vary between aerobically and anaerobically growing cells, anaerobic efficiency was calculated to be 24.0%, which was approximately 50% of its aerobic counterpart (47.9%). Fenchel and Finlay (5) previously showed, theoretically and experimentally, that the gross growth efficiency of anaerobic protozoa was 10%, which was approximately 25% that of the aerobic protozoa. This value was estimated using ATP production via glycolysis and substrate-level phosphorylation (*i.e.*, fermentation). The values obtained for *S. clinomigrationis* were higher than their estimates, suggesting that *S. clinomigrationis* uses the electron transport chain, such as nitrate and fumarate respiration, to generate ATP under anoxic conditions (6). Transmission electron microscopic observations showed no significant differences between the numbers of mitochondria and structures of mitochondrial cristae in *S. clinomigrationis* cells cultured under oxic and anoxic conditions (19). The preservation of mature mitochondria and generation of ATP by nitrate respiration in anoxic water was previously reported in the facultative anaerobic ciliate, *Loxodes* sp. (7). However, some fungal species may grow using ammonia fermentation under anoxic conditions (24, 26). The facultative anaerobic fungus, *Fusarium oxysporum*, retains mature mitochondria under oxic conditions, but has fewer mitochondrial cristae under anoxic conditions (26). The degeneration of cristae under anoxic conditions indicates that the electrochemical proton gradient system of aerobic respiration is dispensable. The systems used to generate ATP in the anaerobic growing cells of *S. clinomigrationis* have not yet been identified. An expression analysis of genes involved in anaerobic respiration and/or an examination of electron acceptor metabolism may provide answers to these questions.

To the best of our knowledge, this is the first study to show the grazing kinetics of (facultative) anaerobic HNF. Thus, we cannot compare the values for bacteria ingestion rates obtained in this study with those of other anaerobic HNF. We examined grazing kinetics in our culture under optimal growth conditions, with various levels of bacterial abundance in the water column of Lake Suigetsu (18). Bacterial abundance in the anoxic layer of the lake was 2.0–8.9×10^6^ cells mL^−1^, and the highest abundance of bacteria (5.0–18×10^6^ cells mL^−1^) was detected in the oxic–anoxic interface layer. These bacterial densities are insufficient to support the maximum growth rate in the oxic water layer. Bacterial abundance was too low for *Imax_anoxi_* and *μmax_anoxi_*, even in the anoxic layer of the lake. Thus, the growth and grazing rate of *S. clinomigrationis* appear to be limited by the density of bacterial prey in all layers of Lake Suigetsu.

In conclusion, the new isolate of *S. clinomigrationis* grew both aerobically and anaerobically within a wide range of growth temperatures and salinities. These results indicate that this isolate has the ability to inhabit the whole water column of Lake Suigetsu, including the saline sulphidogenic hypolimnion and oxic low-saline epilimnion, throughout the year. However, its growth and grazing kinetics suggest that growth is restricted by low bacterial abundance in the lake. HNF predators, larger protists, including ciliates, and metazoan zooplankton, such as rotifers and copepods, were not observed in the anoxic layer of the lake (18), indicating that HNF predation by ciliates was negligible. Therefore, it may be advantageous for *S. clinomigrationis* to grow in anoxic water below the oxycline of Lake Suigetsu by escaping from the epilimnion, in which predation pressure may be high. Further studies are needed in order to investigate the spatiotemporal distribution of *S. clinomigrationis* in Lake Suigetsu.

## Supplementary Information



## Figures and Tables

**Fig. 1:**
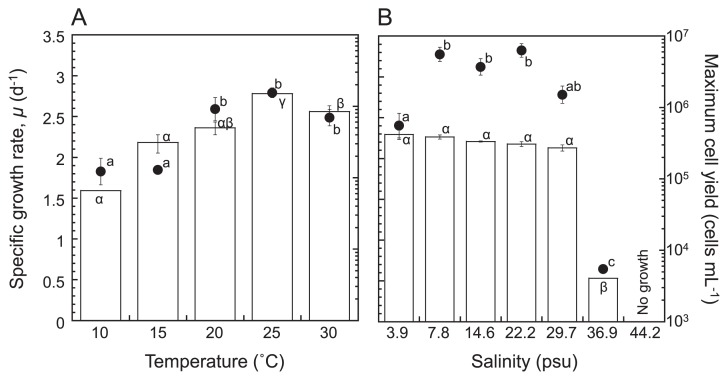
Specific growth rate (*μ*; filled circles) and maximum cell yield (white bars) of *S. clinomigrationis* as a function of temperature (A) and salinity (B) under oxic conditions. Error bars represent the standard error of the mean (*n*=3). Columns marked with the same letter (English letters for specific growth rate and Greek letters for maximum cell yields) do not differ significantly at α=0.05.

**Fig. 2:**
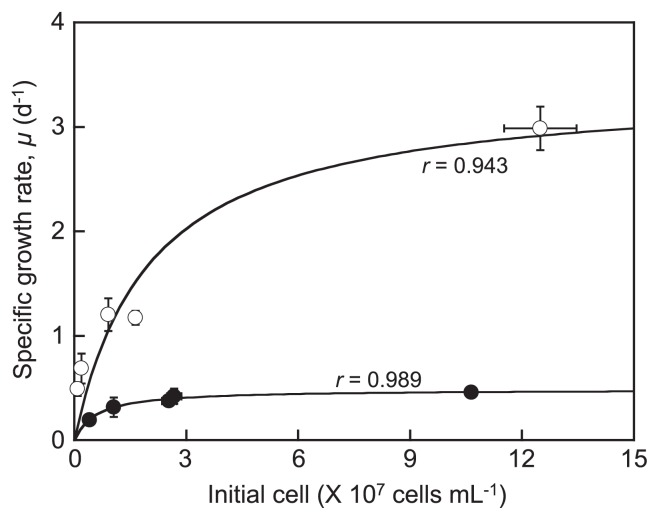
Specific growth rate (*μ*) of *S. clinomigrationis* as a function of the bacterial prey density under oxic (empty circle) and anoxic conditions (filled circle). Horizontal and vertical bars indicate the standard error of the mean (*n*=3). The correlation coefficients (*r*) of Monod curves are shown.

**Fig. 3:**
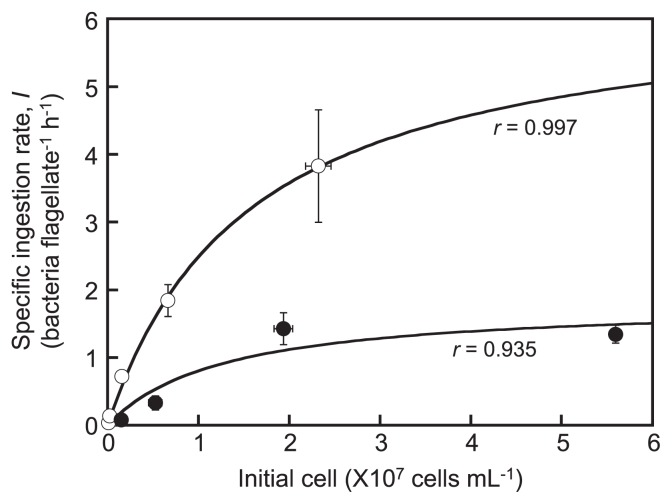
Specific ingestion rate (*I*) of *S. clinomigrationis* as a function of the density of bacterial prey under oxic (empty circle) and anoxic conditions (filled circle). Horizontal and vertical bars indicate the standard error of the mean (*n*=3). The correlation coefficients (*r*) of Michaelis-Menten curves are shown.
