# Supplementary material for: Growth and Grazing Kinetics of the Facultative Anaerobic Nanoflagellate, Suigetsumonas clinomigrationis
Source: Microbes Environ. 2017 Feb 11;32(1):80–3. doi: 10.1264/jsme2.ME16113 (PMC5371079; doi:10.1264/jsme2.ME16113)
Supplement: Supplementary file 1 [file 32_80_s1.pdf]

Supplementary Information for

"Growth and Grazing Kinetics of the Facultative Anaerobic Nanoflagellate, *Suigetsumonas clinomigrationis*"

Ryuji Kondo and Takahiko Okamura

Supplementary Materials and Methods

Strains

In the present study, we used *S. clinomigrationis* NIES-3647, isolated from the dissolved-oxygen depleted water just below the oxic–anoxic interface of the meromictic Lake Suigetsu (2). A facultative anaerobic *Arcobacter* sp. strain co01 was isolated from the polyxenic culture of *S. clinomigrationis* by plating on 1.5% (w/v) agar plate of YE100oxi medium (2). Phylogenetic analysis of 16S rRNA gene of this bacterial strain showed that the nearest neighbour of the isolate was the *Arcobacter cloacae* strain SW28-13 (Accession no. NR_117570), with sequence similarity of 99% (data not shown). The 16S rRNA gene sequence of *Arcobacter* sp. co01 was deposited in DDBJ under accession number LC198182.

Establishment of monoxenic culture

To avoid the effect of changes in the bacterial community on the physiology of *S. clinomigrationis* in the polyxenic culture, a monoxenic culture of *S. clinomigrationis* with *Arcobacter* sp. was established as follows. The polyxenic culture of *S. clinomigrationis* was filtered through a 10-μm membrane filter (Nuclepore, Whatman, Tokyo, Japan) without suction to remove the bacterial frocks and filamentous bacteria. The filtrate was then passed through a 2.0-μm pore membrane filter (Nuclepore, Whatman) without suction, and the filter was washed with autoclaved lake water three times. The culture on the filter was transferred to a plastic tube. An aliquot (1 mL) of the *S. clinomigrationis* culture was inoculated into a 9-mL culture of *Arcobacter* sp. containing streptomycin at a final concentration of 100 μg mL^{-1} and incubated at 20 °C in the dark. After 2 days of incubation, 1 mL of the culture was again inoculated into a 9-mL culture of *Arcobacter* sp. containing streptomycin. These inoculations were repeated four times until the establishment of monoxenic culture. The monoxenic culture was checked by epifluorescence microscopic examination of 4'-6-diamidino-2-phenylindole (DAPI)-stained bacterial cells and sequence analysis of PCR-amplified 16S rRNA gene using the DNA extracted from the culture as a template. The monoxenic culture of *S. clinomigrationis* and *Arcobacter* sp. was maintained in the YE100ppm medium (2) at 20 °C in the dark.

Growth temperature and salinity

S. clinomigrationis was cultured in YE100oxi medium for one week at 20 °C in the dark until the late exponential growth phase. *Arcobacter* sp. was cultured in YE1000oxi medium (1000 mg L^{-1} yeast extract in the water taken from oxic–anoxic interface of Lake Suigetsu) for three days. The *Arcobacter* sp. cells were collected by centrifugation at 2,330 × *g* for 15 min at room temperature and resuspended in diluted (4×) Daigo's artificial seawater SP (Nihon Pharmaceutical, Tokyo, Japan). Then, 1 mL of each culture was inoculated into 18 mL of YE100oxi medium to obtain the initial *S. clinomigrationis* density of 10^2 to 10^3 cells mL^{-1} and the initial *Arcobacter* sp. density of *ca.* 10^8 cells mL^{-1}. This bacterial density did not limit the growth of *S. clinomigrationis*. Triplicate cultures were incubated at 10, 15, 20, 25 and 30 °C in the dark under oxic conditions. Aliquots of the cultures were subsampled at appropriate intervals, fixed by adding 1/10 volume of 10% (v/v) glutaraldehyde in 0.1 M sodium cacodylate buffer (SCB) and stained with DAPI. Stained cells of *S. clinomigrationis* were collected on 0.8-μm black Nuclepore membrane filter (Whatman). The cells on the filters were counted using an Olympus BX51 epifluorescence microscope (3). Specific growth rates of *S.*

clinomigrationis were assessed using the changes in cell counts in the exponential growth phases. *Arcobacter* sp. cells were also counted using the DAPI-staining method to check for the changes in cell numbers during incubations.

To assess the levels of salinity required for the growth of *S. clinomigrationis*, we used the Daigo's artificial seawater SP. The salinities were 3.9, 7.8, 14.6, 22.2, 29.7, 36.9 and 44.2 psu; the solutions were prepared by serial dilution of the artificial seawater. Pre-cultured *S. clinomigrationis* and *Arcobacter* sp. were inoculated into the artificial seawater solutions at the initial densities of *ca.* 10^4 cells mL^{-1} and *ca.* 10^8 cells mL^{-1}, respectively. Triplicate cultures were incubated at 25 °C in the dark under oxic conditions and were subsampled every 12 h. The cells of *S. clinomigrationis* and *Arcobacter* sp. were counted in the same way as in the growth temperature experiment.

Numerical response

Arcobacter sp. was aerobically cultured in YE1000ppm medium (lake water in the YE1000oxi medium was replaced with diluted (4×) artificial seawater) at 25 °C for 3 days. The *Arcobacter* sp. cells were washed in the sterile diluted (4×) artificial seawater and centrifuged (2,330 × *g* for 15 min) three times. The cells were suspended in the diluted seawater at a final concentration of *ca.* 10^8 cells mL^{-1}. A 20-mL pre-culture was started by addition of 2 mL of a stock culture of *S. clinomigrationis* to the *Arcobacter* suspension and grown for 7 days at 25 °C in the dark. The pre-culture (0.2 mL) of *S. clinomigrationis* was inoculated at final densities of *ca.* 10^3 cell mL^{-1} into triplicate oxic 20 mL of (4 ×) diluted artificial seawater containing various densities of *Arcobacter* sp. (final densities from 10^6 to 10^8 cells mL^{-1}). The *Arcobacter* sp. cultures were incubated in the YE1000ppm medium, at 25 °C in the dark for 5 days and washed three times as stated above. The carry-over of *Arcobacter* sp. in the experimental culture from pre-culture of *S. clinomigrationis* was less than 10^6 cells mL^{-1}. The anaerobic pre-culture and experimental culture conditions were similar to those in the aerobic cultures, except for the presence of 3 µM Na_2S and 1 mg L^{-1} of resazurin in the media and artificial seawater. In this case, the incubation period for the pre-culture of *S. clinomigrationis* was 10 days. After inoculation, the cultures were incubated at 25 °C in the dark. Anaerobic conditions were checked by the absence of colour changes of resazurin in the media. The cells of *S. clinomigrationis* were counted every 12 h using the method described above. The specific growth rate (μ) was calculated using the regression analysis of the linear portion of the graph associated with the exponential growth phase. The relationship between the specific growth rates of *S. clinomigrationis* and the initial bacterial densities fitted a hyperbolic function of a form of Monod kinetics; $\mu = \mu max \times B / (K\mu + B)$, where μmax is the maximum specific growth rate, $K\mu$ is half-saturation constant, and B is the initial density of *Arcobacter* sp. The parameters μmax and $K\mu$ were calculated using the Michaelis–Menten model in KaleidaGraph programme. These parameters were expressed as μmax_{oxi} and $K\mu_{oxi}$ under oxic conditions, and μmax_{anoxi} and $K\mu_{anoxi}$ under anoxic conditions.

Functional response

For the analysis of the grazing kinetics of *S. clinomigrationis*, the pre-culture and experimental cultures were prepared in the same way as for the growth kinetics experiment. Bacteria ingestion rates were estimated by the short-term tracer method using 0.5-µm diameter fluorescently labelled beads, FLBeads (Fluoresbrite® Microparticles, Polysciences, PA, USA). The FLBeads were added to the each tube at *ca.* 10% of the inoculated *Arcobacter* sp. density. In the preliminary experiments, the total number of FLBeads ingested by *S. clinomigrationis* increased linearly within 60 min of incubation (data not shown). Thus, the test tubes were incubated for 60 min at 25 °C in the dark. After incubation, a 1-mL subsample was taken and fixed immediately with an equal volume of ice-cold 4% (v/v) glutaraldehyde buffered with SCB to stop the egestion of surrogates taken into the food vacuoles of the nanoflagellate (4). To account for FLBeads adsorbed on the cell surfaces of *S. clinomigrationis*, a zero-time control was taken and fixed as described above. The fixed samples were filtered on 0.8-µm black Nuclepore membrane filters and stained with primulin solution (250 µg mL^{-1}; 1). At least 100 cells in each sample were inspected using epifluorescence microscopy under UV excitation. The FLBeads in the food vacuoles were counted under blue-light excitation (1). The specific ingestion rate (I; bacteria $flagellate^{-1}$ h^{-1}) of *S. clinomigrationis* was calculated as follows: $I = (G_f \times N_b) / (P \times N_f \times T)$, where G_f is the number of FLBeads ingested by the flagellate, and N_b and N_f are the total bacterial and FLBead densities,

respectively. *P* is the number of the *S. clinomigrationis* cells, and *T* is the incubation time. The relationship between the specific ingestion rates and the initial bacterial densities fitted to a hyperbolic function $I = Imax \times B / (Ki + B)$, where *Imax* is the maximum specific ingestion rate, *Ki* is the half-saturation constant and *B* is the initial density of *Arcobacter* sp. The parameters *Imax* and *Ki* were calculated using Michaelis-Menten model in the KaleidaGraph programme. These parameters were expressed as $Imax_{oxi}$ and Ki_{oxi} under oxic conditions, and $Imax_{anoxi}$ and Ki_{anoxi} under anoxic conditions.

Statistical analysis

Bartlett's test of equal variance was followed by one-way ANOVA or Kruskal-Wallis one-way ANOVA for the data on growth rates and maximum cell yields under different conditions of temperature and salinity. Differences were assessed by post-hoc Bonferroni's multiple comparison test and the threshold of significance was set at $p = 0.05$ for all the analyses.

References for supplemental information

1. Okamura, T., Y. Mori, S. Nakano, and R. Kondo. 2012. Abundance and bacterivory of heterotrophic nanoflagellates in the meromictic Lake Suigetsu, Japan. Aquat. Microb. Ecol. 66:149-158.
2. Okamura, T. and R. Kondo.2015. *Suigetsumonas clinomigrationis* gen. et sp. nov., a novel facultative anaerobic nanoflagellate isolated from the meromictic Lake Suigetsu, Japan. Protist. 166: 409-421.
3. Porter, K.G. and Y.S. Feig. 1980. The use of DAPI for identifying and counting aquatic microflora. Limnol. Oceanogr. 25:943-948.
4. Sanders, R.W., K.G. Porter, S.J. Bennett, and A.E. DeBiase. 1989. Seasonal patterns of bacterivory by flagellates, ciliates, rotifers, and cladocerans in a freshwater planktonic community. Limnol. Oceanogr. 34:673-687.

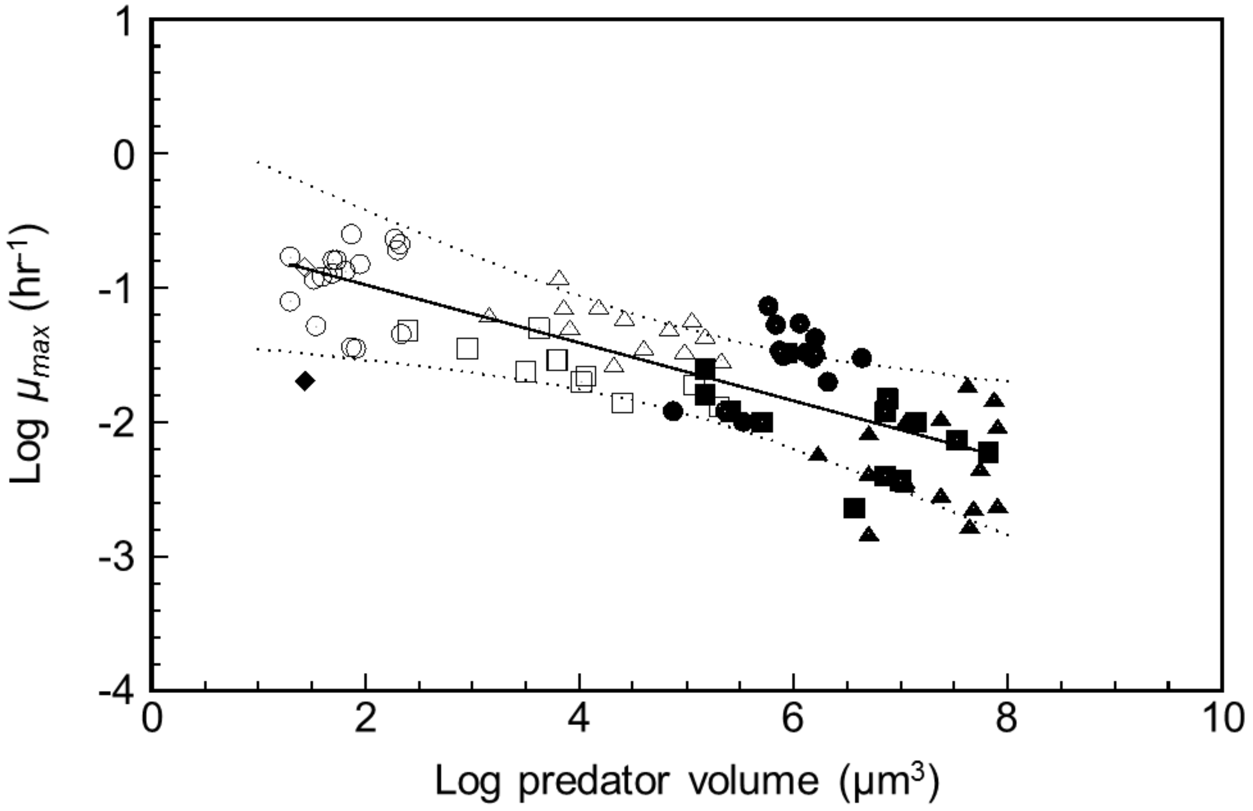

Fig. S1. Maximum specific growth rate (μ_{max}) as a function of cell volume for different phagotrophic plankton taxa. Nanoflagellates (○), dinoflagellates (□), ciliates (△), rotifers (●), meroplankton larvae (■), cladocerans (▲) and *S. clinomigrationis* (◊ for aerobic growth, ♦ for anaerobic growth). A solid line and dotted lines indicate an overall regression and 95% confidence intervals, respectively. The source of data: Hansen et al. (8).
